# Atypical lateralization of visuospatial attention can be associated with better or worse performance on line bisection

**DOI:** 10.1007/s00429-024-02822-3

**Published:** 2024-06-22

**Authors:** Esteban Villar-Rodríguez, Tatiana Davydova, Lidón Marin-Marin, César Avila

**Affiliations:** 1 Neuropsychology and Functional Neuroimaging, Universitat Jaume I, Castelllón de La Plana, Spain; 2 Department of Psychology, University of York, York, UK

**Keywords:** Functional hemispheric lateralization, Language production, Visuospatial attention, Cognitive performance, Landmark task, Left-handers

## Abstract

The causal and statistical hypotheses diverge in determining whether the lateralization of language function in one cerebral hemisphere entails the lateralization of visuospatial function in the opposite hemisphere. Additionally, it remains unclear if the atypical segregation of these functions could influence cognitive performance. This study addresses these questions by examining the hemispheric lateralization of visuospatial attention during a line bisection judgement (landmark) task in three groups of healthy non-right-handed individuals with different language production segregations: left (typical), ambilateral (atypical), and right (atypical). Consistent with the causal hypothesis, results indicate that the groups with left and right language lateralization primarily utilize the opposite hemisphere for visuospatial attention. The ambilateral group, however, displays a pattern compatible with an independent segregation, supporting the statistical hypothesis. Behavioral analyses reveal that atypical lateralization of visuospatial attention (non-right) can lead to either better or worse performance during the landmark task, depending on the specific pattern. Bilateral organization is associated with reduced overall accuracy, whereas the left segregation results in improved performance during the most challenging trials. These findings suggest the existence of diverse pathways to lateralization, akin to either the causal or statistical hypothesis, which can result in cognitive advantages or disadvantages.

## Introduction

The lateralization of cognitive functions in different hemispheres is one of the fundamental characteristics of the human brain (Hartwigsen et al. 2021). Functions that are perhaps most strongly lateralized are language in the left hemisphere and visuospatial attention in the right hemisphere, as evidentiated by unilateral lesion syndromes (Broca 1861; Mesulam 1981) and functional magnetic resonance imaging (fMRI) (Fink et al. 2001; Mazoyer et al. 2014). Language functions are mainly controlled by two separate hubs located in the left hemisphere: Broca’s area (critically involved in expressive language) and Wernicke’s area (more associated with comprehension processes) (for a review, see Price 2012). Both areas are connected by two different pathways: a dorsal one (articulatory and syntactic processing, supported by the left arcuate fasciculus) and a ventral one (semantic processing, supported by the left inferior occipitofrontal and uncinate fasciculi) (Hickok and Poeppel 2007; Friederici 2012). Parallelly to this left dorsal pathway, a right frontoparietal network has been critically implicated in line bisection (landmark) and other visuospatial tasks (Seydell-Greenwald et al. 2019), to the point of describing the visuospatial neglect syndrome after its right damage (Vossel et al. 2010; Molenberghs et al. 2012). However, despite the extensive research on the hemispheric specialization of these two functions, questions about the mechanisms underlying their hemispheric segregation and its associated cognitive consequences remain without a definitive answer.

To what extent is the cerebral lateralization of these two functions dependent on each other? There are two opposing theories on this aspect. The causal hypothesis suggests that the brain segregation of both functions is interdependent, so the location of one of them in one hemisphere implies the location of the other in the opposite hemisphere (Kosslyn 1987; Hellige 1990). In contrast to this view is the statistical hypothesis, which postulates that hemispheric specialization is supported by independent processes, and thus the hemispheric segregation of these cognitive functions in different hemispheres responds to a purely probabilistic phenomenon (Bryden et al. 1983). Studies using fTCD and fNIRS have tested these hypotheses in right-handed samples—mostly typically lateralized—of 20 to 52 individuals (Dorst et al. 2008; Lust et al. 2011; Rosch et al. 2012; Jia et al. 2022). All four studies failed to find a correlational association between the lateralization of language and visuospatial attention (*r* ranging from 0.01 to 0.21), thus supporting the statistical hypothesis.

A different approach, however, is to investigate visuospatial segregation in left-handed individuals, who present a higher representation (approximately 22–27%) of atypical—non-left—language lateralization (Mazoyer et al. 2014). If the causal hypothesis is correct, we should observe a shift to the left hemisphere in the lateralization of visuospatial attention among these atypical individuals. The results in this population have been, however, hard to interpret, so a detailed disclosure of previous results is warranted. Flöel et al. (2001, 2005) found using fTCD that 10 left-handers with a strongly right-lateralized language did lateralize visuospatial functions (measured via the landmark task) to the left hemisphere, but collateralized segregation of both functions in the right hemisphere were also possible (especially among weakly lateralized or ambilateral participants). A different fTCD study, on the other hand, did not find any cases of reversed lateralization of visuospatial processing using a spatial memory task in 8 individuals with right-lateralized language (Whitehouse and Bishop 2009). In the fMRI study by Badzakova-Trajkov et al. (2010) in 155 individuals of different handedness, a negative correlation was found between the lateralization of both functions (landmark task) at the group level (*r* =  − 0.18), although all combinations of dissociated and collateralized segregations were present at the individual level at a distribution compatible with the statistical hypothesis. In contrast, the study by Cai et al. (2013) described a reversal of visuospatial lateralization (landmark task) in all 13 strongly right-lateralized participants (*r* =  − 0.54) in a sample with no ambilateral individuals for language, although no significant relation was found among the typicals (*r* =  − 0.05). In a similar vein, after examining 142 right-handers and 151 left-handers, Zago et al. (2016) revealed that the hemispheric complementarity between both functions (sentence production and landmark tasks) was only evident in strong left-handers (regression slope =  − 8.7). Finally, the sample in Gerrits et al. (2020a, b)—consisting of 39 typicals and 24 atypicals (including ambilaterals) who completed a landmark task—presented an incidence of reversals (3 out of 4) incompatible with the statistical hypothesis, but still not applicable to all participants, and thus also not fully compatible with the causal hypothesis. Similarly, in Vingerhoets et al. (2018) they also found that 3 out of 4 atypicals presented a reversal during the landmark task.

Taken altogether, it would seem that the principles of segregation behind these two functions: (1) concur with the statistical hypothesis among typically lateralized right-handers; but (2) seem to be the result of both dependent and independent processes among atypically lateralized left-handers, as demonstrated by hemispheric complementarity being more frequent than predicted by the statistical hypothesis (but still far from causality). As for the different results in left-handed and right-handers, it should be noted that left-handedness could have a direct impact on hemispheric lateralization strength besides increasing the incidence of atypical lateralizations (Johnstone et al. 2021). Regarding the second conclusion, employed methodologies have raised some concerns—fTCD vs. fMRI, inclusion vs. exclusion of weakly lateralized, lateralization grouping cut-offs, and sample size—in the validity and joint interpretation of the results (see Cai et al. 2013; and Badzakova-Trajkov et al. 2016). Also, a new hypothesis—the segregation bias model—has recently been proposed to conciliate these two classic theories (Gerrits 2024). Briefly, this model postulates that functional lateralization is independently biased towards one hemisphere in a function-by-function basis (akin to the statistical hypothesis), but that a certain percentage of the population present a global reversal of these biases (including handedness), resulting in the existence of complete segregation reversals among some left-handers. Relevant to both the classic and new models—and to the current study—the presence of ambilateral participants has been suggested as a contributing factor to data favoring the statistical hypothesis among left-handers (Cai et al. 2013). This idea is further supported by hemispheric complementarity being present only among the most left-handed (Zago et al. 2016), who happen to represent the most lateralized atypical individuals (Mazoyer et al. 2014). But no study to date has directly tested the validity of this claim.

Another open question is the behavioral consequences of the different phenotypes of lateralization. Hemispheric specialization is suggested to offer several advantages when facing complex cognitive processes, as it allows for the parallel processing of diverse aspects of stimuli (e.g., linguistic and visuospatial) while also reducing the level of required interhemispheric transmission, promoting a faster and more efficient processing (Ringo et al. 1994; Rogers 2000; Vallortigara and Rogers 2020). Unfortunately, studies exploring these suggestions among healthy individuals are scarce. In a—mostly typical—right-handed sample of 16 participants, Everts et al. (2009) described a linear relationship between strength of lateralization and cognitive performance in the language (*r* = 0.58; lateralization of language production; verbal IQ) and spatial (*r* =  − 0.62; lateralization of visual search; Rey Figure and Corsi Block Test) domains. However, a different study by Lidzba et al. (2011) in 36 right-handers found an increase in verbal IQ as left lateralization of language comprehension (story task) decreased (*r* = 0.49). When considering atypical lateralization phenotypes, Mellet et al. (2014) found via fMRI that 37 individuals with weak lateralization of language (including left-handers and right-handers) performed worse—partial *η*^*2*^ = 0.03—in tests measuring verbal (vocabulary, fluency, reading span, listening span, and rhyme judgment), verbal memory (words and pseudowords recall) and spatial (mental rotation, maze test, Corsi Block Test, and Raven matrix) components. However, a previous fTCD study by Knecht et al. (2001) challenged this notion, failing to find an association between lateralization of language and performance variables related to academic performance in a sample including 31 ambilaterals and 31 right-lateralized. Also noteworthy are the fMRI studies supporting the ‘hemispheric crowding’ hypothesis; that is, that worse cognitive performance is related to an atypical functional segregation (i.e. language and visuospatial processing together in the left or right hemisphere), rather than a totally reversed segregation (Powell et al. 2012; Vingerhoets et al. 2018; Gerrits et al. 2020b). So, in conclusion, further research is needed to better elucidate the role of hemispheric lateralization of these two functions in cognitive ability.

In the current study, we used fMRI to analyze the relationship between hemispheric lateralization of language production (verb generation task) and visuospatial attention (landmark task) in a relatively big sample of non-right-handed individuals, preselected to be rich in right-lateralized and ambilaterals for language. Our two objectives were: (1) to test the association between the lateralization of language and visuospatial attention in both strongly lateralized and weakly lateralized persons; and (2) to explore the effects of different lateralization phenotypes on performance during the visuospatial task. We hypothesized that: (1) strongly lateralized would segregate both functions in different hemispheres, whereas ambilaterals would present no clear pattern (Cai et al. 2013; Zago et al. 2016); and (2) ambilaterals would perform worse during the landmark task (Ringo et al. 1994; Rogers 2000; Mellet et al. 2014; Vallortigara and Rogers 2020).

## Methods

### Participants

Seventy-nine (79) participants were included in this study, seventy-one (71) of whom pertained to a cohort described in a previous study (Villar-Rodríguez et al. 2023). All participants were left-handed (n = 68) or mixed-handed (n = 11) according to the Edinburgh Handedness Inventory/EHI (Oldfield 1971). EHI was scored by computing the Laterality Quotient or LQ, according to the formula (R − L/R + L)*100 (LQ lower or equal to − 50 was considered left-handed, and LQ between − 50 and + 50 was considered mixed-handed) (Szaflarski et al. 2002). Note that an alternative EHI scoring according to the method proposed by Bryden (1977) is available at the published database (see Data Availability statement). The hemispheric language lateralization of participants was calculated following the completion of an fMRI verb generation task based on which the participants were categorized as left-lateralized (n = 41), right-lateralized (n = 17), or ambilateral (n = 21) for language. We used this task for two reasons: (1) we have demonstrated its potential for determining language lateralization in the inferior frontal gyri in both presurgical patients and healthy participants (Sanjuán et al. 2010); and (2) for consistency with our previous studies on language lateralization among left-handers (Villar‐Rodríguez et al. 2020; Villar-Rodríguez et al. 2023), as well as future studies involving presurgical patients. There were no statistically significant differences between the groups in terms of age (*F* = 1.60; *P* = 0.21), sex (*χ2* = 4.88; *P* = 0.78), or EHI-LQ (*F* = 1.04, *P* = 0.36). A slightly significant difference (*F* = 3.13, *P* = 0.049) was detected in fluid intelligence (measured via WAIS-IV matrix reasoning subtest; Wechsler 2012) between the left-lateralized and right-lateralized groups (Bonferroni’s pair-wise *P* = 0.053). Descriptive statistics of the lateralization groups can be found in Table 1.

**Table 1:** Descriptive statistics for the left-lateralized, right-lateralized and ambilateral groups according to hemispheric lateralization of language

	Left-lateralized (*n* = 41)	Ambilateral (*n* = 21)	Right-lateralized (*n* = 17)
Sex	20 male, 21 female	11 male, 10 female	7 male, 10 female
Age (years)	22.6 ± 4.81	24.76 ± 5.74	24.71 ± 7.22
EHI (LQ)	− 72.16 ± 31.88	− 80.12 ± 26.96	− 83.12 ± 25.49
WAIS-IV (score)	12.49 ± 2.13	11.71 ± 2.22	11 ± 2

^a^Age, EHI and WAIS-IV are expressed as mean ± standard deviation

Participants were recruited following a screening fMRI session in which language lateralization was roughly assessed using real-time data from the verb generation task (BrainWave software, GE HealthCare Technologies Inc.). This screening procedure was responsible for the high proportion of right-lateralized and ambilaterals (see the subsection ‘Cohort acquisition’ for more details). None of the participants reported any history of head injury resulting in the loss of consciousness, or psychiatric or neurological disorders. All participants signed an Informed Consent Form prior to participating in the study, following a protocol approved by the Universitat Jaume I Ethics Committee. All methods and procedures were carried out in accordance with the approved guidelines and current regulations.

### Cohort acquisition

Participation in the current study was advertised on university announcement boards and in the local media. Every healthy non-right-handed person over 16 years old was invited to participate in an fMRI session (S1). In this S1 session, general demographic variables were acquired, including in-house batteries that evaluate bilingualism history and musical experience. Standardized tests were also administered for: bilingual switching (BSWQ), sensitivity to punishment and reward (SPSRQ), and musical reward (BMRQ). Music-related variables have been the focus of a different study not yet published (note that the sample used in Villar-Rodríguez et al. [2020] is an entirely different cohort). Bilingualism-related variables are relevant for a different study we are still working on. Sensitivity to punishment/reward was acquired to complement a different line of research focused on personality traits. An audiometry test was also performed to ensure normal hearing.

During the fMRI session of S1, participants completed: (1) 3D and DTI structural acquisitions; (2) resting-state (eyes closed); (3) verb generation task (Spanish), (4) comprehension task (Spanish); (5) verb generation task (Valencian/Catalan, completed only by bilinguals); and (6) word listening task (Spanish).

We used real-time scanner software to roughly determine if the participant was typically or atypically lateralized during the verb generation task. If the participant was considered potentially atypical (crearly right-lateralized or probably ambilateral), they were invited to participate in a different fMRI session (S2) to take place in the future. Potentially typical participants were also invited to participate in S2, until matching the amount of potentially atypicals who agreed to participate in S2. We prioritized inviting potentially typical participants whose age and sex roughly matched those of the potentially atypical participants.

In total, 174 participants completed S1. However, due to time constraints and/or technical reasons, not all participants were able to complete all fMRI sequences (all 174 completed at least the verb generation task). 90 participants (45 potentially atypical, 25.86% of all participants, which is in line with published incidences of atypical lateralization among left-handers) were invited to participate in S2.

In S2, standardized tests were administered for: general intelligence (WAIS-IV matrix reasoning subtest), schizotypy traits (SPQ), autistic spectrum traits (AQ), and dyslexic traits (PROLEC-SE-R word reading subtest). Additional behavioral tests involving auditory and language processing were also administered (spoonerisms and second phoneme detection). Data regarding schizotypy traits, autistic spectrum traits, and dyslexic traits have been explored in a previous publication (Villar-Rodríguez et al. 2023). Auditory and language processing tests are currently being analyzed as part of a different study. General intelligence has been used as a control variable in all studies deriving from this cohort that have explored cognitive performance in any way (such as the accuracy during the landmark task in the current study, or the SSRT during the stop-signal task in Villar-Rodríguez et al. 2023).

During the fMRI session of S2, participants completed: (1) 3D and FLAIR structural acquisitions; (2) stop-signal task (Villar-Rodríguez et al. 2023); (3) landmark task (current study), and (4) reading task.

In total, 90 participants completed S2. However, due to time constraints and/or technical reasons, not all participants were able to complete all fMRI sequences (hence the sample differences between this study and Villar-Rodríguez et al. 2023). Also, in both this study and Villar-Rodríguez et al. (2023), different participants had to be excluded due to low engagement during the relevant tasks.

### Verb generation task

Expressive language function was measured by way of fMRI verb generation task (Sanjuán et al. 2010) that consisted of a block design paradigm with activation and control conditions. During the activation condition, participants were presented with a series of nouns and were requested to say the first verb that came to mind when seeing each word. During the control condition, participants were asked to read aloud visually presented pairs of letters. The task was administered using E-prime 2.0 (https://pstnet.com/products/e-prime) and included 6 activation and 6 control blocks. Each block lasted 30 s with each stimulus duration of 1500 ms and with a blank inter-stimulus interval of 1500 ms. Prior to performing the task in the scanner, each participant received detailed instructions on performing the task and completed a practice trial that lasted 2 min. Stimuli were presented using MRI-compatible googles (VisuaStim Digital, Resonance Technology Inc.) and verbal responses were recorded with a noise-cancelling microphone (FOMRI III + , Optoacoustics Ltd.) to ensure task compliance.

### Landmark task

**Fig. 1:**
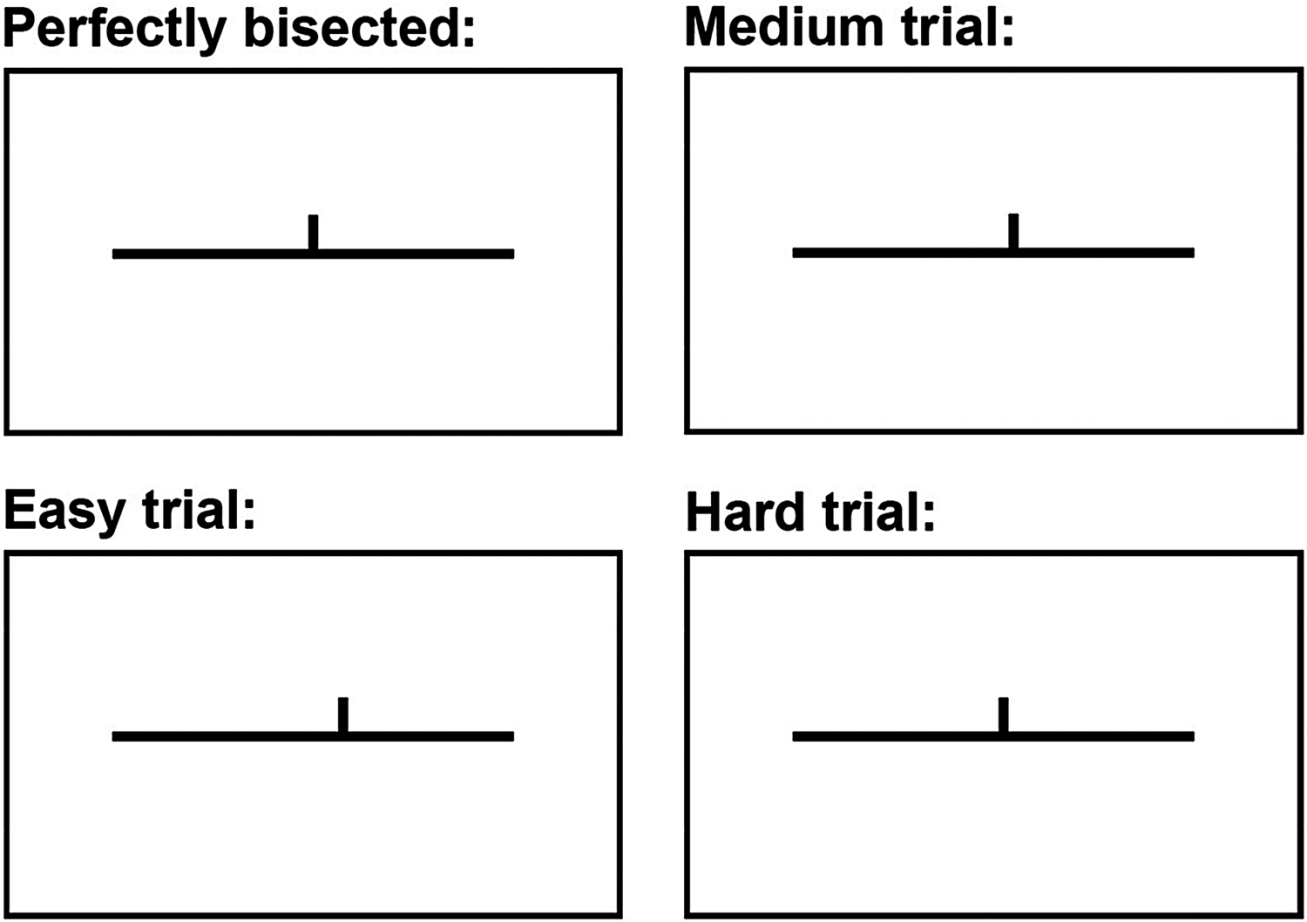
Showcase of the different trial types presented during the ‘activation’ condition of the landmark task. Participants had 1.815 s to respond whether the presented line was perfectly bisected or not

Visuospatial processing was examined by fMRI landmark task (Ciçek et al. 2009). During this task, participants were presented with a series of horizontal lines pre-bisected with a short vertical line and were required to respond by pressing the index button on the left response grip if the line was bisected correctly (task condition) and the thumb button if not. In this condition, the lines were bisected correctly in 40% of the trials or deviated to the right or left of the midline by 2.5% (hardest difficulty), 5% (medium difficulty) or 7.5% (easiest difficulty) of the line’s length, each deviation presented in 10% of the trials (see Fig. 1). During the control condition, the participants were required to respond whether the presented horizontal line and the bisection mark touched (index button) or not (thumb button). In this condition, the lines were touching in 40% of the trials, and not touching in 60% of the trials. These task parameters are identical to those described in Ciçek et al. (2009), which were also used by Cai et al. (2013) and Gerrits et al. (2020b), differing only slightly from Badzakova-Trajkov et al. (2010) and Zago et al. (2016). The task was administered using E-prime 2.0 (https://pstnet.com/products/e-prime) and included 7 activation and 7 control blocks. Each block lasted 22 s and started with a 4-s instruction, followed by a 215 ms blank inter stimulus interval and a 1.6-s presentation of a total of 12 line images. To avoid the use of the screen center as reference during the activation trials, and to ensure that participants correctly engaged in visuospatial processing, line images were not centered on the screen but slightly tilted to the left or right, alternating between trials. Prior to performing the task in the scanner, each participant received detailed instructions and completed a practice trial that consisted of 1 activation and 1 control block. Stimuli were presented using MRI-compatible goggles (VisuaStim Digital, Resonance Technology Inc.), and goggles-adapted corrective lens were available to ensure perfect visual perception for all participants. During the task, data on accuracy (% of correct responses) across all types of trials was recorded to measure task performance. Responses were registered with an MRI-compatible response grip (ResponseGrips, NordicNeuroLab). All participants responded using their left hand.

### Image acquisition

Images were acquired on a 3 T General Electric Signa Architect magnetic resonance imaging (MRI) scanner using a 32-channel head coil. All slices were acquired in the sagittal plane. A 3D structural MRI was acquired for each subject using a T1-weighted magnetization-prepared rapid gradient-echo sequence (TR/TE = 8.5/3.3 ms; flip angle = 12; matrix = 512 × 512 × 384; voxel size = 0.47 × 0.47 × 0.5). For the functional images, a gradient-echo T2*-weighted echo-planar imaging sequence was used to acquire 150 functional volumes for the verb generation task (TR/TE = 2500/30 ms; flip angle = 70; matrix = 64 × 64 × 30; voxel size = 3.75 × 3.75 × 4). A different gradient-echo T2*-weighted echo-planar imaging sequence was used to acquire 185 functional volumes for the landmark task (TR/TE = 2000/30 ms; flip angle = 70; matrix = 64 × 64 × 27; voxel size = 3.75 × 3.75 × 4.5).

### Image processing

The processing of the functional images was carried out using the Statistical Parametric Mapping software package (SPM12; Wellcome Trust Centre for Neuroimaging, London, UK) and MATLAB (version R2018b, MathWorks, Natick, MA). The default pipeline was followed during preprocessing steps that included: (a) aligning the functional data to the AC‐PC plane by using the anatomical image; (b) head motion correction, realigning and reslicing the functional images to the mean functional image; (c) coregistration of the anatomical image to the mean functional image; (d) re‐segmentation of the anatomical image; (e) spatial normalization of the functional images to the MNI (Montreal Neurological Institute, Montreal, Canada) space with a 3 mm3 resolution; followed by (f) spatial smoothing with a 4-mm full-width-at-half-maximum (FWHM) Gaussian kernel. The general linear models (GLM) for both the verb generation task and the landmark tasks were defined for each participant by contrasting activation > control blocks. For both tasks, the BOLD (Blood‐Oxygen‐Level‐Dependent) signal was estimated by convolving each task’s block/trial onsets with the canonical hemodynamic response function (HRF). Six motion realignment parameters were included as nuisance regressors, and a high‐pass filter (128 s) was applied to the contrast images to account for low-frequency drifts.

### Individual functional lateralization and group distribution

Functional lateralization for each task was assessed by obtaining the Laterality index (LI) using the bootstrap method implemented in the LI-toolbox for SPM12 (Wilke and Lidzba 2007). The LI is computed by calculating the proportion of activation differences between the two hemispheres for each individual subject. For the verb generation task, we explored the LI of the areas of the inferior frontal gyrus responsible for language production, specifically, pars opercularis and pars triangularis (Price 2012). For the landmark task, the LI calculation centered on the posterior areas involved in visuospatial attention during this task (Fink et al. 2001; Ciçek et al. 2009; Cavézian et al. 2012; Cai et al. 2013; Zago et al. 2016), specifically: supramarginal gyrus, angular gyrus, and the superior division of the lateral occipital cortex (Harvard–Oxford atlas). Masks were defined using the maximum probability Harvard–Oxford atlas (Frazier et al. 2005; Makris et al. 2006; Desikan et al. 2006; Goldstein et al. 2007), and were fitted to our functional images via the mask pre-processing step in LI-toolbox. The LI ranges from + 100 (total left functional lateralization) to -100 (total right functional lateralization), thus providing information about the direction and degree of hemispheric lateralization during a given task. The participants were thus categorized as left-lateralized if their LI was > 40, right-lateralized if their LI was <  − 40, and ambilateral if their LI was in between − 40 and 40. We used ± 40 as a cut-off point based on previous findings that emphasized the importance of lateralization strength when grouping individuals (Mazoyer et al. 2014; Labache et al. 2020). Considering that one of our objectives was to disentangle the differences between strongly lateralized and weakly lateralized, we opted for a cut-off that maximized that contrast by ensuring the strong lateralization of both the left-lateralized and right-lateralized groups.

### Statistical analyses

A series of analyses were performed to test the hypothesis of crossed dominance of the parietal network involved in visuospatial processing in individuals with atypical language lateralization. First, a Kruskal–Wallis test was computed to check if significant differences existed in landmark LI between the left, right and ambilateral groups according to language. Post hoc pair-wise comparisons were calculated using the Dunn test. Next, Spearman’s correlation was used to study the linear relationship between the LIs for the verb generation and the landmark tasks.

Voxel-wise whole-brain activations during the landmark and verb generation tasks were also explored in relation to language and visuospatial lateralization groups, respectively. One-sample *t*-tests were computed to describe the activation pattern during the ‘activation > control’ condition across the whole sample (voxel-wise *P* < 0.001; FWE cluster-corrected at *P* < 0.05). Then, voxel-wise two-sample* t*-tests were used to examine activation differences between the groups with left, right, and ambilateral language lateralization according to the verb generation task and the landmark task (voxel-wise *P* < 0.001; FWE cluster-corrected at *P* < 0.05).

We also studied behavioral performance during the landmark task in relation to hemispheric lateralization. First, accuracy (%) when correctly detecting the bisected lines was compared across all groups using two separate ANOVA designs (one for language-based groups, and one for visuospatial-based groups), including age and fluid intelligence (WAIS-IV score) as covariates of control. Then, two separate repeated-measures ANOVAs (one for language-based groups, and one for visuospatial-based groups) were also computed exploring the trials requiring the detection of incorrectly bisected lines. These models included difficulty (easy, medium, hard) as within-subject factor, lateralization group (left, right, or ambilateral) as between-subject factor, and the accuracy rate as a dependent variable. Age, fluid intelligence (WAIS-IV score), and accuracy rate during correctly bisected trials were used as covariates of control.

## Results

### Visuospatial attention shifts its lateralization depending on language lateralization

**Fig. 2:**
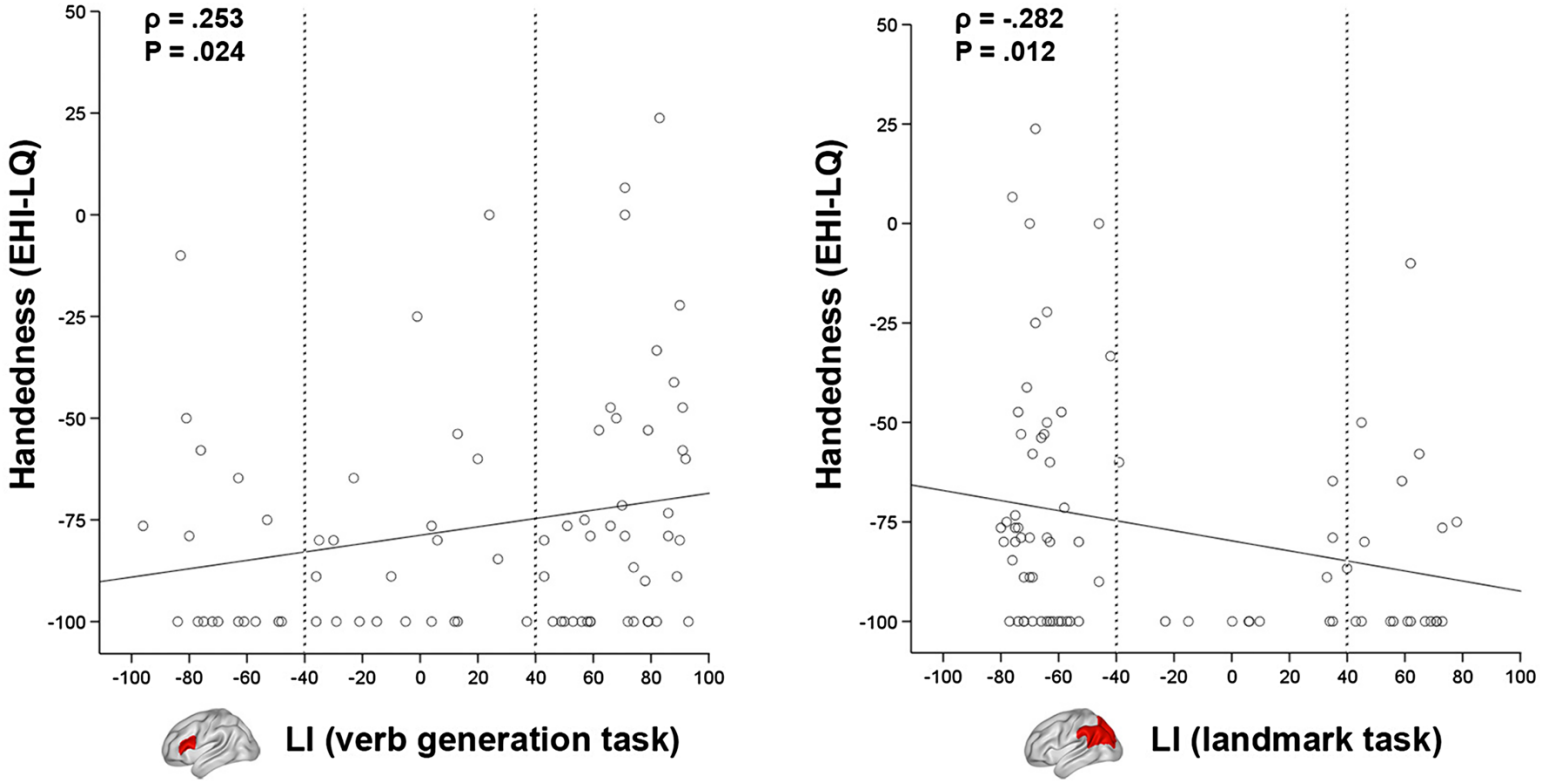
Relationship between handedness (EHI-LQ) and Laterality Index (LI) during the verb generation task (left) and the landmark task (right). Negative LI values indicate rightward lateralization, whereas positive LI values indicate leftward lateralization. Lower EHI-LQ indicates stronger left-handedness. Note that all participants were non-right-handed, so the lower scores should be interpreted as mixed-handedness

We used the landmark and verb generation tasks to assess the hemispheric lateralization of visuospatial attention and language production, respectively, via the calculation of Laterality Indexes (LI). The distribution of participants across lateralization groups according to both LIs can be found in Table 2. Interestingly, significant correlations were found across the whole sample between the handedness score (EHI-LQ) and the verb generation LI (*ρ*_*77*_ = 0.253, two-tailed *P* = 0.024; Fig. 2) plus landmark LI (*ρ*_*77*_ =  − 0.282, two-tailed *P* = 0.012; Fig. 2). That is, stronger left-handedness was associated to both rightward lateralization of language production and leftward lateralization of visuospatial attention.

**Table 2:** Distribution of hemispheric lateralization groups for the verb generation and landmark tasks

	Verb generation task		
	Left-lateralized (*n* = 41)	Ambilateral (*n* = 21)	Right-lateralized (*n* = 17)
Landmark task			
Left-lateralized (*n* = 19)	7.3% (3)	23.8% (5)	64.7% (11)
Ambilateral (*n* = 12)	4.9% (2)	19% (4)	35.3% (6)
Right-lateralized (*n* = 48)	87.8% (36)	57.2% (12)	0% (0)

^a^Verb generation groups are used as reference for the displayed percentages

**Fig. 3:**
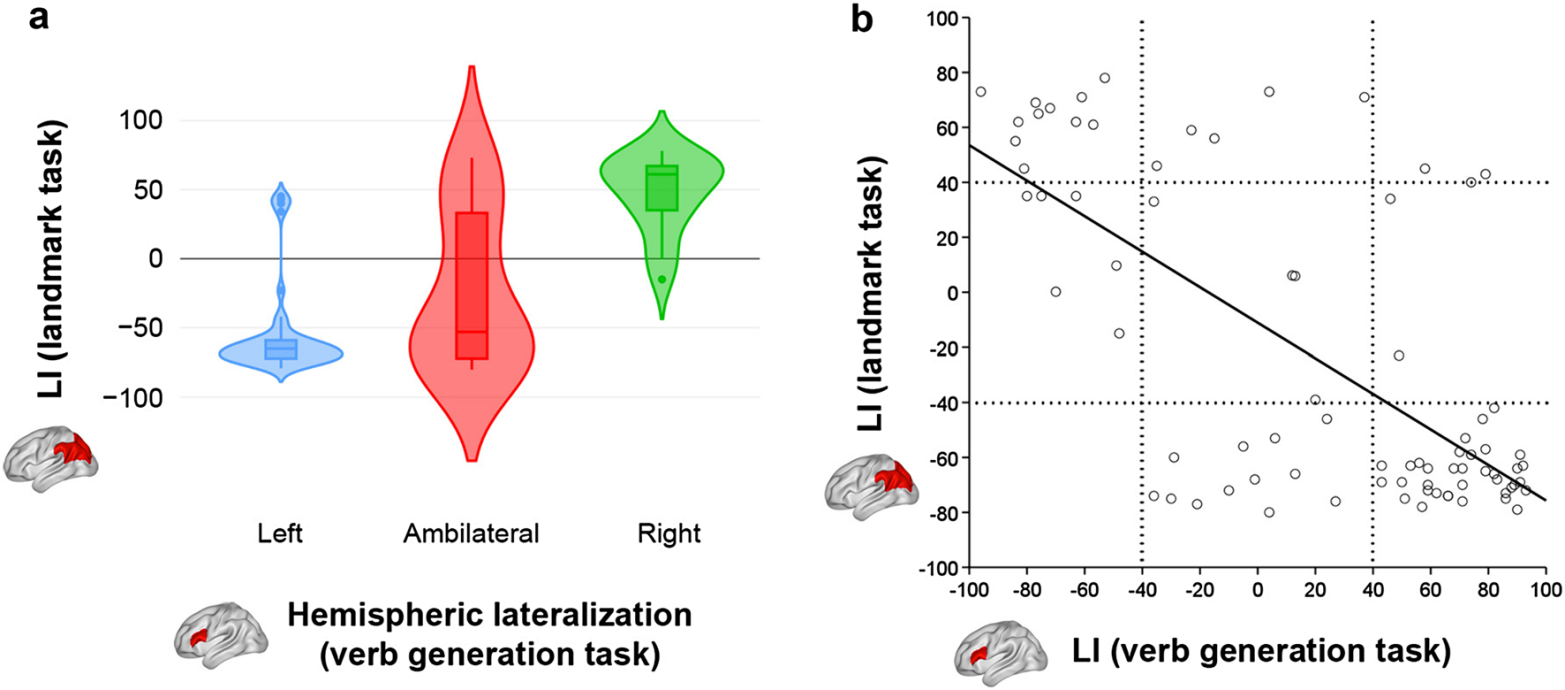
Relationship between hemispheric lateralization of language production and visuospatial attention. **a** Violin plot depicting the group differences in landmark LI according to verb generation groups (*H*_2_ = 31.19, *P* < 0.001). **b** Correlation between the LIs of the verb generation and landmark tasks (*ρ*_79_ =  − 0.554, two-tailed *P* < 0.001, *R*^*2*^ = 0.47). Negative values indicate rightward lateralization, whereas positive values indicate leftward lateralization

As evidenced by a significant Kruskal–Wallis test (*H*_2_ = 31.19, *P* < 0.001), the group right-lateralized for language differed in its landmark LI (median = 61) when compared to the left-lateralized (median =  − 65) and ambilateral groups (median =  − 53). Left-lateralized and ambilateral groups, however, did not significantly differ (Post hoc pair-wise comparisons: left vs. right *P* < 0.001; right vs. ambilateral *P* < 0.001; left vs. ambilateral *P* = 0.087) (Fig. 3a). Moreover, this complementarity was also evident continuously. A Spearman’s correlation analysis between both LIs indicated a strong inverse relationship across the whole sample (*ρ*_77_ = -– 0.554, two-tailed *P* < 0.001). (Fig. 3b).

**Fig. 4:**
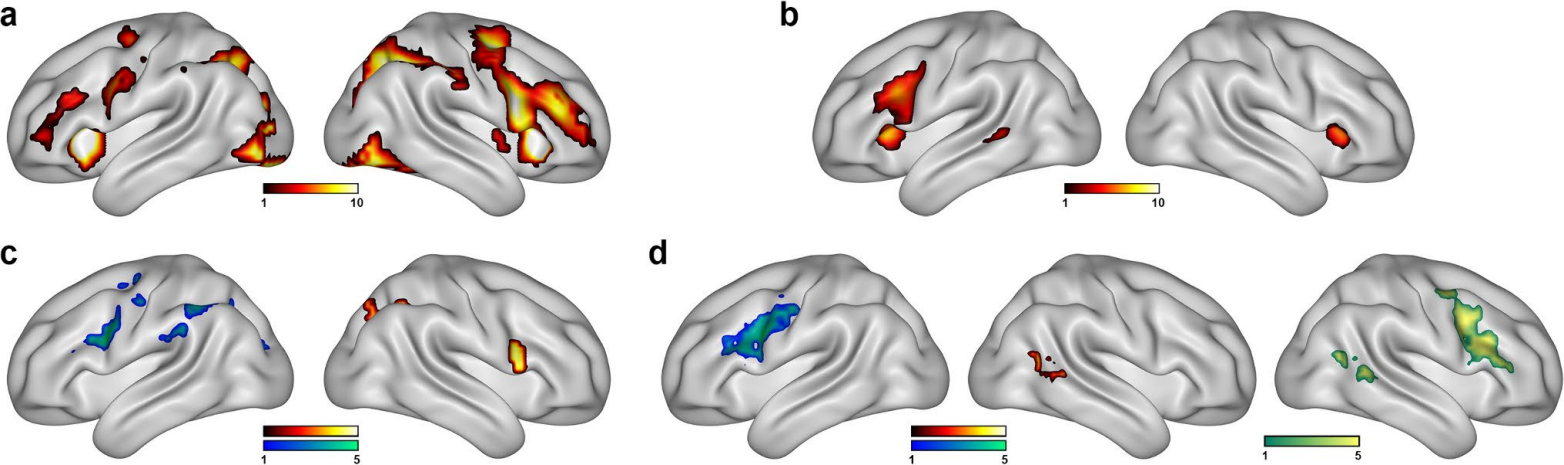
Whole-brain analyses of the landmark and verb generation tasks. **a** Results of the one-sample *t* tests in all participants showing the brain areas involved during the landmark task (activation > control; voxel-wise *P* < .001; FWE cluster-corrected at *P* < 0.05; N = 79). **b** Results of the one-sample *t* tests in all participants showing the brain areas involved during the verb generation task (activation > control; voxel-wise *P* < 0.001; FWE cluster-corrected at *P* < .05; N = 79). **c** Results of the two-sample *t* tests showing increased activation during the landmark task in left-lateralized (hot colors, right image) and right-lateralized (cold colors, left image) groups according to language (activation > control; voxel-wise *P* < .001; FWE cluster-corrected at *P* < .05; N = 41 left-lateralized and 17 right-lateralized). Ambilaterals (N = 21) presented no significant differences when compared to left or right-lateralized. **d** Results of the two-sample *t* tests showing increased activation during the verb generation task in left-lateralized vs. right-lateralized (hot colors, middle image), right-lateralized vs. left-lateralized (cold colors, left image), and ambilaterals vs. right-lateralized (green colors, right image) groups according to visuospatial processing (activation > control; voxel-wise *P* < 0.001; FWE cluster-corrected at *P* < 0.05; N = 19 left-lateralized, 12 right-lateralized and 48 ambilaterals). Ambilaterals presented no significant differences when compared to left-lateralized. Color bars represent *t* value

We also explored the group-wise cerebral activations during the landmark and verb generation tasks. Whole-brain one-sample *t*-tests confirmed that the landmark task activated an extensive attentional network comprised of anterior cingulate gyrus, superior frontal gyrus, and superior plus inferior parietal gyrus (including supramarginal and angular gyrus), as well as the superior lateral occipital cortex (voxel-wise *P* < 0.001; cluster-wise FWE-corrected at *P* < 0.05) (Fig. 4a). Verb generation task activated the left inferior frontal gyrus, left middle temporal gyrus, bilateral insula and bilateral supplementary motor area (Fig. 4b). We additionally computed two-sample *t*-tests to compare whole-brain activation patterns during the landmark and verb generation tasks between left, right, and ambilateral lateralization groups according to language production and visuospatial attention, respectively (Fig. 4c and d). In the landmark task, no significant differences were found between the ambilateral group and the left- or right-lateralized groups for language. However, when comparing both segregated groups, the group with the left language lateralization presented increased rightward activation during the landmark task, while the right-lateralized group showed an increased left hemispheric activation of different components of the dorsal attentional network, including inferior frontal gyrus, precentral gyrus extending into the frontal eye fields, supramarginal gyrus, and superior lateral occipital cortex (voxel-wise *P* < 0.001; cluster-wise FWE-corrected at *P* < 0.05). In the verb generation task, the right-lateralized for visuospatial processing presented an increased activation in the left inferior frontal gyrus and left precentral gyrus, while the left-lateralized showed increased activation in the right middle temporal gyrus. Ambilaterals, when contrasted to right-lateralized, exhibited increased activation in the right inferior frontal gyrus and right middle temporal gyrus. No significant differences were found between ambilaterals and left-lateralized for visuospatial processing.

### Influence of hemispheric lateralization on task accuracy

All groups showed a high accuracy during the landmark task when detecting correctly bisected lines, whether grouped by lateralization of language production (mean ± standard deviation: left: 88.4 ± 9.5%; right: 89.4 ± 6.2%; ambilateral: 86.8% ± 11%) or visuospatial attention (left: 88.7 ± 7.4%; right: 87.3 ± 10.4%; ambilateral: 91.3 ± 6.8%), with no significant differences detected (language groups: *F*_2,76_ = 0.051, *P* = 0.604; visuospatial groups: *F*_2,76_ = 1.22; *P* = 0.301).

**Fig. 5:**
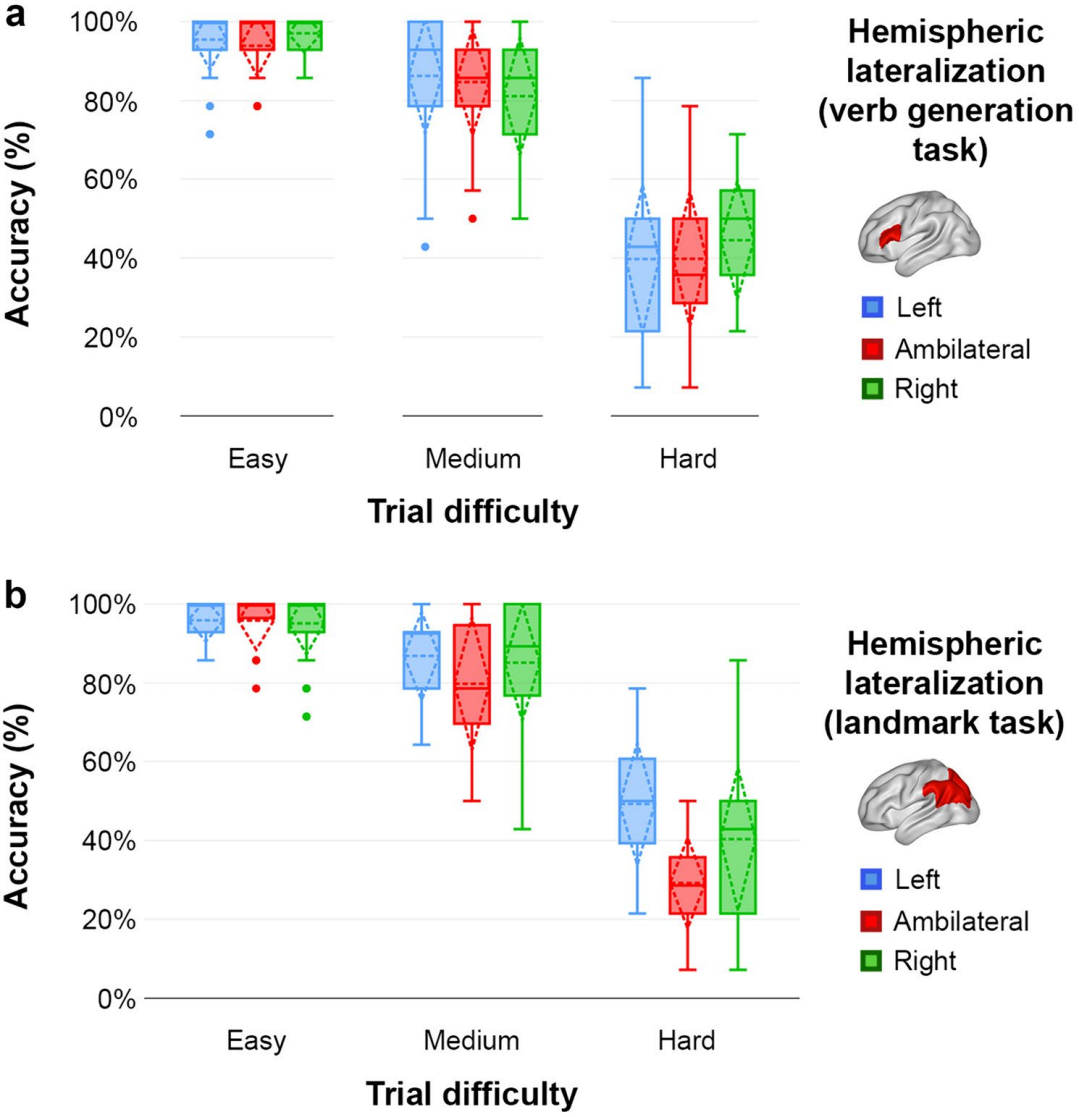
Hemispheric lateralization and behavioral performance (% of correct responses) during the different trials of the landmark task. **a** According to language (verb generation task) hemispheric lateralization. **b** According to visuospatial attention (landmark task) hemispheric lateralization. Plots depict mean ± standard deviation (dashed lines), median ± inter-quartile range (box), and data range (whiskers)

In a more fine-grained analysis, we used repeated-measures ANOVAs to compare the accuracy of the different lateralization groups when the line was not correctly bisected, while also considering trial difficulty. Hemispheric lateralization of language was not found to be associated in any way with performance differences during the landmark task (Fig. 5a). Lateralization of visuospatial attention, on the other hand, produced a significant main effect of Group (*F*_2,73_ = 3.21, *P* = 0.046), hinting at ambilaterals (68% global accuracy) performing worse across all difficulties than left-lateralized (77.1% global accuracy; pair-wise *P* = 0.013) and right-lateralized (73.7% global accuracy; pair-wise *P* = 0.077) (Fig. 5b). Also, the Group × Difficulty interaction reached significance (*F*_4,146_ = 2.59, *P* = 0.039), revealing an advantage of left-lateralization of visuospatial attention over right-lateralization (pair-wise *P* = 0.034) and ambilaterality (pair-wise *P* = 0.002) in the most difficult trials. That is, left-lateralization during the landmark task resulted in the highest accuracy on the hardest trials. It should be noted that the comparison between ambilaterals and right-lateralized participants during these trials was also close to significance in favor of the later (pair-wise *P* = 0.066).

## Discussion

The current study investigated the relationship between hemispheric lateralization of language production (verb generation task) and visuospatial attention (landmark task) in a diversely lateralized sample of non-right-handed participants. The main conclusions derived from the obtained results are that left- and right-lateralized groups for language present predominantly segregated visuospatial processing in the opposite hemisphere, while the ambilateral group for language showed a more diffuse pattern. Importantly, we also found that hemispheric lateralization of visuospatial attention (but not language production) was associated with the visuospatial performance during the landmark task. The ambilateral group presented the worst overall accuracy, whereas the left-lateralized group—that is, the inverted pattern of control—exceled in the hardest trials.

In consonance with prior fMRI studies, the landmark task elicited activations in the inferior and middle frontal gyri, anterior insular cortex, frontal eye fields, anterior cingulate gyrus, inferior and superior parietal lobule, and lateral occipital cortex (Fink et al. 2001; Ciçek et al. 2009; Cavézian et al. 2012; Cai et al. 2013; Zago et al. 2016). But most importantly, we demonstrated that an atypical right hemisphere lateralization of language production was accompanied by a leftward increase during the landmark task in the inferior frontal gyrus, frontal eye fields, inferior parietal lobule, and lateral occipital cortex. Thus, hemispheric dominance of visuospatial attention seems to tilt towards the left hemisphere in these individuals. This is further supported by the significant differences in visuospatial lateralization (measured via a posterior LI) between the different groups according to language lateralization (assessed through an anterior LI). We also found a strong inverse correlation between both LIs across all participants. Taken altogether, these findings align with the causal hypothesis of lateralization (postulating that a complementary relationship exists in the hemispheric segregation of certain cognitive functions) and add to previous evidence in left-handed population (Flöel et al. 2001, 2005; Cai et al. 2013; Zago et al. 2016; Gerrits et al. 2020b). The ambilateral group for language, however, did not conform to this hypothesis. Rather than complementing its ambilateral control for language with an ambilateral control for visuospatial processing, this group showed a pattern similar to left-lateralized individuals but in a more diffuse manner (see Fig. 3a in Results). Most ambilateral individuals still presented a (typical) right-lateralization of visuospatial attention, although cases of (atypical) ambilaterality and left-lateralization were notably more frequent. This fits with the clusterization of language lateralization by Mazoyer et al. (2014), which showcases significant differences between strongly-atypical (akin to our right-lateralized group) and ambilaterals. Noteworthy, we also replicated previous reports of an association between stronger left-handedness and stronger rightward language lateralization (Knecht et al. 2000; Mazoyer et al. 2014), as well as a new association with stronger leftward visuospatial lateralization.

It is essential to note that the previously discussed findings are group-wise results. At the individual level, 3 participants presented a strong collateralization (LI > 40) of both functions in the left hemisphere, and weak lateralization of visuospatial attention (LI between 40 and − 40) was frequent among those who were right-lateralized for language (6 out of 17 individuals). These cases do not seem to conform to the causal hypothesis and, no matter how strong the group-wise correlation was between the lateralization of both functions, it impedes us from stating that individual data unequivocally support the causal hypothesis. In our opinion, this divergence may stem from various reasons. Firstly, our previous report comparing language production and inhibitory control (a function strongly dependent on the right inferior frontal cortex) suggested that the degree of hemispheric complementarity might be influenced by the amount of cerebral crowding (Villar-Rodríguez et al. 2023). In that study, we failed to find a single case of collateralization. So, the more hemispherically homotopical the functions are, the higher the ‘competition’ is for cerebral space, and thus the more prominently hemispheric complementarity manifests (as discussed by Vingerhoets 2019). In the case of language production and visuospatial attention, this overlap is less marked, resulting in more frequent cases of collateralization. In fact, Badzakova-Trajkov et al. (2016) and Cai et al. (2013) already suggested that innate predispositions to certain cerebral asymmetries might not be as present among non-right-handers—under the light of the Annett (2002) and Mcmanus (1985) classic genetic models of lateralization and handedness—thus allowing crowding effects to drive cerebral organization in a complementary manner. However, current molecular genetics have failed to support these models—hinting at complex and polygenetic factors, with little to no overlap between handedness and cerebral lateralization—and thus these assumptions must be taken with caution (for a review, see Vingerhoets 2019).

Another possibility we must consider is that not all non-right-handers may share the same developmental factors for their handedness. In that case, we could speculate that the non-right-handers who deviate from the causal hypothesis might be influenced by a different set of genetic and epigenetic factors. This may potentially make certain left-handed individuals less susceptible to complementary processes and more similar to right-handers in regards to functional segregation (Dorst et al. 2008; Badzakova-Trajkov et al. 2010; Lust et al. 2011; Rosch et al. 2012; Jia et al. 2022). Or it could also be understood under the *mens inversus totalis* (‘inverted mind’) proposal by Vingerhoets (2019), further integrated within the segregation bias model by Gerrits (2024). This hypothesis postulates that, even if independent processes drive hemispheric lateralization, complementarity could still arise in certain individuals after an initial symmetry-breaking event, resulting in both left-handedness and a reversed segregation of functions (the so-called ‘inverted minds’ or reversed typical functional segregation). Regarding our results, a similar case could be made of strongly atypical left-handers (mostly composed of ‘inverted minds’) and ambilateral left-handers (no ‘inverted minds’), which would tentatively explain why the first seem to present causal complementarity, while the later does not.

We found significant differences in performance during the landmark task according to its pattern of hemispheric lateralization. Although all groups performed similarly when detecting correctly bisected lines, their performance differed when they had to detect slight deviations from midline. The ambilateral group—that is, those individuals with a weak lateralization of visuospatial attention—were less accurate regardless of trial difficulty. This fits well with the proposed computational advantage of hemispheric segregation in situations requiring fast processing (Ringo et al. 1994; Rogers 2000; Vallortigara and Rogers 2020), as participants had only 1.8 s to respond in each trial. Similarly, previous results in the BIL&GIN—the largest to date fMRI database of atypically lateralized participants for language—showed that the ambilateral group presented a worse execution on diverse cognitive tests measuring spatial processing, verbal working memory, and language performance (Mellet et al. 2014). Remarkably, in contrast to that study, we directly observed this effect on both the lateralization of visuospatial attention and the performance of the task used to measure it. This finding is also consistent with Gerrits et al. (2020a, b) and Vingerhoets et al. (2018)—but not the replication study by Gerrits and Vingerhoets (2023)—who revealed that left-handed persons with a significant deviation from the cerebral segregation predicted by the causal hypothesis (i.e. atypical segregations) exhibit poorer cognitive performance. Taken altogether, current and previous findings mostly indicate that an ambilateral segregation of cognitive functions might be associated with a worse performance.

A second intriguing behavioral result showed a higher accuracy during the hardest trials (deviation from midline by 2.5% of the line’s length; see Fig. 1 in Methods) when visuospatial attention was left-lateralized. As to why there is an advantage to visuospatial attention when it is segregated in the left hemisphere, we can only speculate. In our previous study (Villar-Rodríguez et al. 2023), we reported an association between rightward lateralization of language and lower accuracy when reading long and unfamiliar words. Perhaps, the structural and functional architecture of the left hemisphere is better suited to control certain cognitive functions than that of the right hemisphere. Interestingly, past studies have consistently showed a higher proportion of left-handers among successful architecture students and faculty (Peterson and Lansky 1974, 1977; Götestam 1990). Taking into account that the completion of architecture studies is associated with better visuospatial skills (Berkowitz et al. 2021), and considering that left-lateralized visuospatial attention is more frequent among left-handers than among right-handers (Zago et al. 2016), our findings could tentatively explain these previous reports.

In summary, different pathways appear to support the development of atypical lateralization of language and visuospatial functions in left-handers, in consonance with the segregation bias model by Gerrits (2024). When one function is strongly lateralized in one hemisphere, the other function is primarily controlled by the opposite hemisphere, consistently with the causal hypothesis (with some exceptions possibly due to the grade of hemispheric crowding). However, weak lateralization of one function is associated with a random segregation of the other, with a slightly higher probability of resulting in a typical lateralization, aligning with the statistical hypothesis. Importantly, in the case of visuospatial attention, these atypical patterns can lead to different behavioral consequences: the ambilateral organization is associated with a poorer performance, whereas the left lateralization results in better performance. It should be noted that previous studies have reached significantly different conclusions about hemispheric complementarity when examining right-handed samples (Flöel et al. 2001, 2005; Dorst et al. 2008; Whitehouse and Bishop 2009; Badzakova-Trajkov et al. 2010; Lust et al. 2011; Rosch et al. 2012; Jia et al. 2022). In fact, left-handedness itself might influence lateralization strength, regardless of atypical lateralization incidence (Johnstone et al. 2021). Future research should aim to describe a sufficiently large group of atypically segregated right-handers to clarify the exact role of handedness in this phenomenon. Behavioral screening procedures might be an efficient way to identify such participants (Gerrits et al. 2020a; Parker et al. 2021).

## Data Availability

The datasets generated and analysed during the current study are available in the figshare repository, 10.6084/m9.figshare.25524145.v1.
